# Characterization of a periodontal-inflammatory microRNA profile during multibracket orthodontic treatment in adolescents

**DOI:** 10.1038/s41598-025-01794-6

**Published:** 2025-06-03

**Authors:** Florian Rolfes, Johannes Heck, Isabelle Riedel, Christian Bär, Boris Schmitz

**Affiliations:** 1 Dental Medical Center ALL DENTE, MVZ, Kamen, Germany; 2 Hannover Medical School, Institute of Molecular and Translational Therapeutic Strategies, Hannover, Germany; 3 Hannover Medical School, Institute for Clinical Pharmacology, Hannover, Germany; 4 R-CUBE Center of Translational Regenerative Medicine, Hannover Medical School, Hannover, Germany; 5 Fraunhofer Institute for Toxicology and Experimental Medicine (ITEM), Hannover, Germany; 6 Department of Rehabilitation Sciences, Faculty of Health, University of Witten/Herdecke, Witten, Germany; 7 DRV Clinic Königsfeld, Center for Medical Rehabilitation, Ennepetal, Germany

**Keywords:** Multi-bracket appliance, Molecular orthodontics, Alveolar bone remodeling, Gingival crevicular fluid, Mechanical loading, Extracellular matrix, Biomarkers, Medical research

## Abstract

This study aimed to identify functional microRNAs (miRNAs) and their respective targets as central regulatory factors of tooth movement during orthodontic treatment. Gingival crevicular fluid (GCF) of 24 adolescent patients (< 18 years) treated with a full-mouth multibracket appliance (MBA; Thermal Copper Nickel Titanium archwire) was analyzed for miRNAs-21, -29b, -34a, -126, -132, -146a, and -221. GCF samples were taken from the second premolar in either jaw using non-invasive sampling before, 7 days, 5 weeks, and 3 months after application of orthodontic force (8 samples per patient). Validated miRNA targets and regulated pathways were identified using the miRTarBase database (release 9.0) and Reactome (version 87). All analyzed miRNAs were consistently detected in the GCF (Ct value < 35) and a moderate to high correlation was found between samples taken from the mandible and maxilla before treatment (r = 0.42 to 0.71, all p ≤ 0.041). All miRNAs showed changes in their expression levels with orthodontic tooth movement compared to baseline (significant time effect, all *p* < 0.001). The general profile indicated an increase in miRNA expression in both jaws with time except for miR-21, which showed reduced levels one week after MBA application (*p* = 0.046). For miR-34, a significant interaction effect was observed (time × jaw, *p* = 0.0396) in that lower levels were found after five weeks and three months of treatment in the mandible compared to the maxilla. The medium to late treatment phase was characterized by an increase in miR-146 and miR-221. Gene signaling pathway analysis suggested regulation of cellular response to stress including hypoxia, matrix reorganization and vascular remodeling. Since the identified miRNA profile was linked to targets involved in the remodeling process of the alveolar supporting apparatus and alveolar bone, GCF-derived miRNAs may represent diagnostic biomarkers to monitor cellular processes during orthodontic tooth movement and potentially optimize individual treatment outcomes.

## Introduction

Mechanical forces during orthodontic treatment induce a multitude of different, time-dependent pathways regulating the complex interplay of bone and periodontal ligament (PDL) resorption and formation. Orthodontic tooth movement has been described to result in independent but simultaneously occurring processes on the pressure side in direction of movement and the contralateral tension side ^1^. The “direct” theory proposes that orthodontic forces directly target bone cells whereas the “indirect” theory suggests that the PDL responds to orthodontic forces, resulting in an activation of osteoclastogenesis by compression and osteoblastogenesis by tension. Recent evidence has led to a new “biphasic” theory suggesting that a catabolic phase accompanied by inflammation is followed by an anabolic phase with osteoclasts regulating the subsequent activation of osteoblasts without site-specificity ^2^. In brief, in the catabolic phase, a perimeter of alveolus degeneration is generated, and the tooth moves in the direction of the orthodontic force, subsequently forming a perimeter of osteogenesis ^2^. When approaching the alveolar bone, disruption of blood circulation with local hypoxia is caused. The volume of the PDL decreases and gingival crevicular fluid is moved from the compressed area via bone canaliculi into the surrounding regions with PDL fibers relaxing. In contralateral direction, PDL fibers tighten and the PDL volume increases with fluid influx. Due to the porosity of the PDL, redistribution of the free fluid phase in the whole periodontium occurs within seconds ^3,4^. This process exposes osteocytes in their lacunae to fluid shear stress, a stimulus that has been suggested as key to activating the osteocyte system ^5^. Regarding to associated bone remodeling, including the resorption of alveolar bone and extracellular matrix, osteoclasts need to be recruited under proinflammatory conditions and angiogenesis is induced for the transport of metabolic products ^6,7^. A crucial force-dependent factor for osteoclast function and enhanced recruitment is the Receptor Activator of Nuclear Factor-κB ligand (RANKL). Since the competitive antagonist of RANKL, osteoprotegerin (OPG), inhibits osteoclastogenesis, the RANKL/OPG ratio determines osteoclast formation and the activation of the remodeling process ^8^. An increase in RANKL and OPG levels can be observed in osteoblasts and PDL fibroblasts as early as three hours after force application ^9–12^. Within twenty-four hours, mechanical stress induces Vascular Endothelial Growth Factor (VEGF), a key angiogenic factor, in fibroblasts and osteoblasts promoting the formation of new blood vessels through proliferation and migration of endothelial progenitor cells ^13,14^. It has been shown that VEGF in osteoblasts is induced by hypoxia through the Hypoxia-Inducible-Factor-1 (HIF-1), which also directly stimulates RANKL expression in PDL fibroblasts ^15,16^. As an inflammatory response to mechanical stress, various cytokines are released from PDL cells, including interleukin (IL)-1α, -4, -6, -10, -12, and Tumor Necrosis Factor-alpha (TNF-α), with increased concentrations within 24 h. Matrix-metalloproteases (MMPs) and the antagonist of MMPs, Tissue Inhibitor of MMP (TIMP), are also released. Together, TNF-α and MMPs, contribute to osteoclast differentiation and, consequently, the direct resorption of alveolar bone ^10,17,18^.

The time-dependent regulation and overall orchestration of the different factors involved is so far only incompletely understood. Recent research has shown that non-coding RNAs such as microRNAs (miRNAs) play a pivotal role in the signal transduction of mechanical forces induced by tooth movement during orthodontic treatments, as they can act locally and in an endocrine manner, potentially orchestrating changes in the remodelling alveolus ^19–21^. miRNAs are small non-coding RNAs (19–25 nucleotides in length) that regulate gene expression at the post-transcriptional level. miRNAs are detectable in virtually all bodily fluids and are expressed in complex networks that enable control of the cellular phenotype ^22^. Studies in the field of pathological inflammatory periodontitis suggest that multiple miRNAs including miRNA-146 and, besides others, miRNA-155 modulate central molecules of bone metabolism such as RANK, RANKL, and OPG via TNFalpha, IL-1beta, and IL-6 regulating osteoclasto- and osteoblastogensis ^23,24^. It has been suggested that mechanical stress, orthodontic forces, and hypoxia in the PDL can lead to the regulation of specific miRNAs involved in bone remodeling and inflammatory processes in the periodontium also under non-pathologic conditions ^25,26^. For example, miR-21 was increased in PDL cells and PDL stem cells of adolescent patients (15–18 years old) during one month of orthodontic treatment, which was associated with increased osteogenesis and alveolar bone remodeling, likely via the IL-12A axis. However, a detailed and long-term profile of miRNAs induced by mechanical forces during orthodontic treatments is currently not available.

Thus, this study aimed to identify a specific set of functional periodontal-inflammatory miRNAs during multibracket orthodontic treatment in healthy adolescent patients. We hypothesized that miRNA alterations would differ over time after treatment initiation depending on associated effectors, providing insight into the involved regulatory pathways.

## Methods

### Study design

This study determined the concentration of miRNAs-21, -29b, -34a, -126, -132, -146a, and -221 in the gingival crevicular fluid (GCF) of adolescent patients (< 18 years) treated with a full-mouth multibracket appliance (MBA). The panel of analyzed miRNAs was the result of an in-depth literature search, considering previous reports on miRNAs affected by orthodontic treatments as well as miRNAs known to be regulated by associated signaling pathways including angiogenesis, hypoxia, etc. ^7,27–36^. Self-reported healthy male and female patients (12–18 years of age) undergoing orthodontic treatment with the insertion of a full-mouth MBA involving more than six bracketed and/or banded teeth per jaw were eligible to participate. All methods were carried out in accordance with relevant guidelines and regulations and written informed consent was obtained from patients and/or their legal guardians. Samples were collected anonymously during routine visits, without documentation of any personal information aligned with the ethics review board of University Witten-Herdeke (reference #115_2020).

### Orthodontic treatment

GCF samples were collected non-invasively from the second premolar in either jaw at four planned time points, before application of orthodontic force, after 7 days, after 6 weeks, and after 3 months of the procedure during routine visits (8 samples per patient). The second premolar was used as the preferred sample site because it normally erupts a year earlier than the canine and the maxillary expansion hyraxes in our protocol were not bound to second premolars. If no second premolar was present, the first premolar was used for collection. Time points for sample collection were chosen to reflect clinical practice, considering the consensus to change archwires or perform orthodontic checks every 6–8 weeks after the start of an orthodontic treatment, also covering longer continuous exposure to initial orthodontic force and the complete implementation of the first archwire ^18,37^. In detail, a 0.014" Thermal Copper Nickel Titanium archwire (Euroform II G&H Orthodontics, Indiana, USA) was used, applying a recovery force of 78 g at mouth temperature. Three types of brackets (22" slot size, MBT prescription) were used. Patients were treated with either a self-ligating MBA (Genius System Metal, ortho Penthin GmbH, Schwanewede, Germany, 4 participants) or one of two conventionally ligated MBAs (SmartTwin, ortho Penthin GmbH, 16 participants; Iconix Aesthetic Braces, American Orthodontics, Wisconsin, USA, 4 participants). The choice of MBA was independent of study participation and solely based on the decision of the legal guardians to cover costs for additional orthodontic service according to German health insurance regulations (e.g. Thermal Copper Nickel Titanium archwires additionally to stainless steel archwires).

### Sample collection and miRNA quantification

Samples were collected during routine visits as described with modification ^27,38^. Before collecting the GCF, patients were instructed to swallow to avoid sample dilution by saliva. The sampling region was isolated vestibular and, in the lower jaw, in the lingual area, using cotton rolls. Tooth were carefully cleared of saliva, especially interdentally, using a dental air syringe. No further cleaning procedures were performed. A PerioPaper (Oraflow Inc., Smithtown, NY, USA) was inserted into the buccal gingival sulcus of a second maxillary as well as a mandibular premolar for 60 s. If no second premolar was present, the first premolar was used for collection. Collection sites were randomly varied, and no specific collection from the pressure or tension side was performed based on the concept that when force is applied to a tooth, the entire alveolus undergoes resorption (catabolic phase) until the force dissipates and if the force is not reapplied, the entire alveolus undergoes formation (anabolic phase). Samples were transferred immediately to 500 μL peqGold TriFast (VWR, Darmstadt, Germany) and frozen. miRNA extraction and quantification were performed at the Institute of Molecular and Translational Therapeutic Strategies (IMTTS) at the Hannover Medical School in a randomized and blinded manner (to avoid systematic extraction errors or handling artifacts). Samples were thawed at room temperature and briefly vortexed. Synthetic Caenorhabditis elegans miR-39-3p (cel-miR-39-3p) was added as an exogenous spike-in control before RNA isolation (1.6 × 10^8^ copies/μL) (Qiagen, Hilden, Germany) as described ^39^. Total RNA was then isolated using a standardized protocol. In brief, 100 μl of chloroform were added and the mixture was thoroughly vortexed. After incubation at room temperature, samples were centrifuged for 5 min (12,000 g, room temperature). An equal volume of isopropanol was added to the supernatant, followed by incubation at − 20 °C for 10 min. After centrifugation, (4 °C, 12,000 g) the supernatant was removed, and the pellet was washed twice by adding 500 μl of 75% ethanol. The RNA pellet was air-dried and dissolved in 15 μL RNase-free water. Samples were stored at − 80 °C until analysis. The isolated RNA (2.5 μl) was reversely transcribed into cDNA using the TaqMan MicroRNA Reverse Transcription (RT) Kit (Applied Biosystems, Darmstadt, Germany) following the manufacturer’s instructions. The RT reaction was performed for 30 min at 16 °C, 30 min at 42 °C, and 5 min at 85 °C, followed by cooling to 4 °C. For RT-qPCR, the cDNA was diluted 1:3 with water, and 2 μL were used in 10 μL qPCR reactions on a Viia7 RT-PCR System (Fisher Scientific, Waltham, Massachusetts, USA) under standard conditions.

The expression levels of hsa-miRNA-21-5p, hsa-miRNA-29b-3p, hsa-miRNA-34a-5p, cel-miR-39, hsa-miRNA-126-3p, hsa-miRNA-132-3p, hsa-miRNA-146a-5p, and hsa-miRNA-221-3p were determined using the corresponding TaqMan miRNA assays (Applied Biosystems) and amplification was performed using the ViiA 7 Real-Time PCR System (Applied Biosystems). Relative miRNA levels were determined by first exporting the raw amplification data to the LinRegPCR (11.0) software which was used to perform QC on amplification to calculate the initial concentration (N0) of a given miRNA per sample ^40,41^. The relative miRNA levels (RQ) were then calculated as RQ = (N0, miRNA-XY/N0, Cel39). The relative miRNA values were log-transformed and normalized to the mean Ct values of the baseline groups.

### Target gene and pathway analysis

Functional miRNA targets were identified using the experimentally validated miRNA-target interaction database miRTarBase ^42^ (https://mirtarbase.cuhk.edu.cn) as described ^43^. The database allows the selection of different in vitro validation methods including reporter assays, western blotting, and quantitative polymerase chain reaction (qPCR), all of which were selected to retrieve a validated list of functional miRNAs. Pathway analysis was performed against Reactome Version 87 (December 2023; human targets ^44^, submitting the identified targets (Supplemental Table 1) to the online analysis tool (https://reactome.org). Reactome provides an overrepresentation analysis using a hypergeometric distribution test that determines whether certain pathways are enriched in the submitted data compared to what is expected by chance. A probability score, corrected for false discovery using the Benjamani-Hochberg method, is provided.

**Table 1:** Mean Ct values by miRNA over all analyzed samples.

miRNA	Assay ID	Mean Ct value (min–max)
hsa-miRNA-21-5p	000,397	19.8 (16.95–25.39)
hsa-miRNA-29-3p	000,413	26.1 (23.39–31.79)
hsa-miRNA-34a-5p	000,426	26.1 (23.04–23.23)
hsa-miRNA-126-3p	002,228	27.5 (22.31–34.24)
hsa-miRNA-132-3p	000,457	26.8 (23.76–32.66)
hsa-miRNA-146a-5p	000,468	24.5 (21.31–31.55)
hsa-miRNA-221-3p	000,524	24.5 (21.89–29. 37)

### Statistical analyses

The statistical analyses were conducted using SPSS, Version 28.0 (IBM, Chicago, USA), and GraphPad PRISM 10.0 (GraphPad Software Inc., La Jolla, USA). Data is presented as mean ± standard deviation (SD) or 95% confidence interval (CI). Data was checked for normal distribution using D’Agostino-Pearson test (Omnibus K2-Test). Differences over time between the upper and lower jaw (interaction effect) were determined using mixed-effects model. Correlation analysis was performed using Pearson correlation coefficient and linear regression. The significance level was set to *p* < 0.05. The calculation of the required sample size (power calculation) was conducted based on a comparable study ^38^ suggesting effect sizes (Cohen’s d) between 1.4 and 2.0, one and four weeks after MBA insertion. With seven miRNAs and a corrected α = 0.007 at a power of 1-β = 0.95 in a repeated-measures ANOVA, a minimum sample size of 23 participants was calculated (G*Power 3.1.9.7).

## Results

Twenty-four patients completed the study protocol. After baseline assessment (T1), mean follow-up sampling time points were as follows. One week (8 ± 2.4 days) after the insertion of the MBA (T2), five weeks (37.1 ± 12.1 days; T3) and three months (91.9 ± 8.0 days; T4) after the insertion of the MBA. Only four GCF samples were missing (two patients missed one appointment) and a total of 188 samples were analyzed. Of note, 99.4% of all analyzed miRNA signals were within the defined detection threshold (Ct value < 35). The mean Ct values of each analyzed miRNA are given in Table 1.

**Fig. 1:**
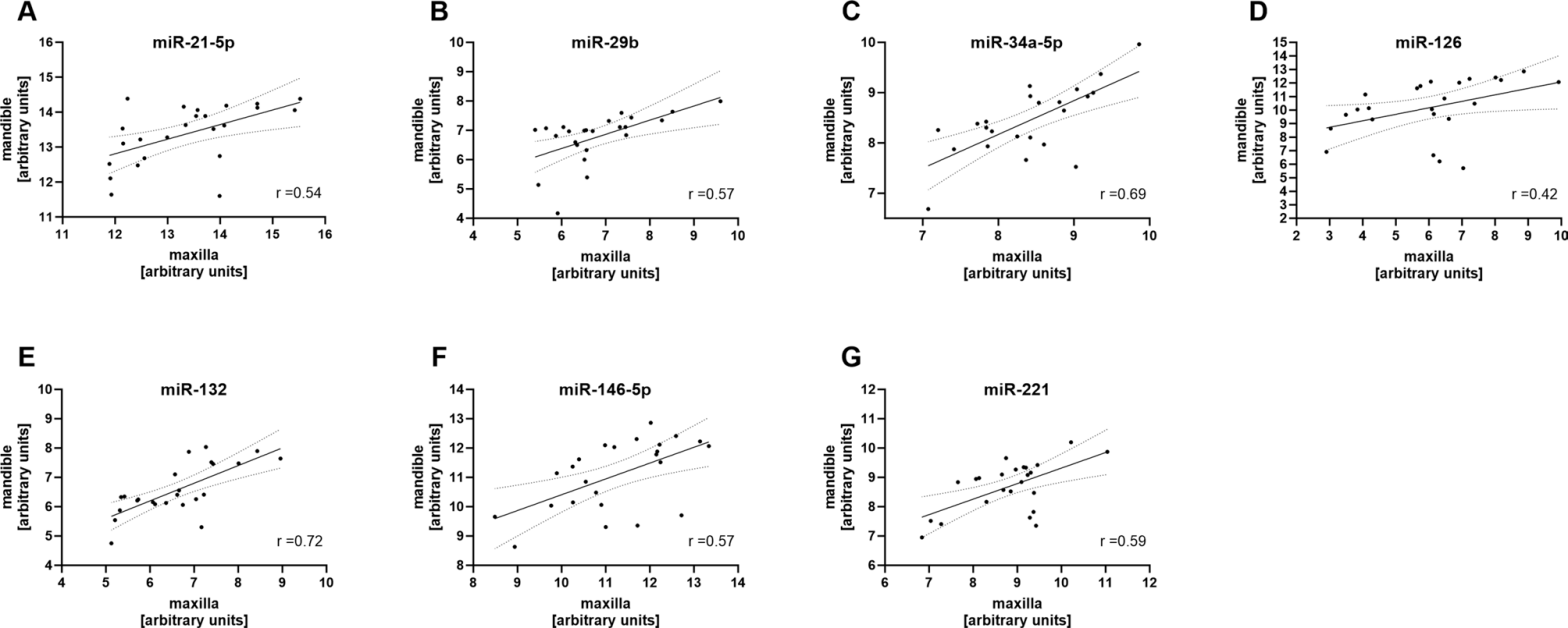
miRNA levels are largely comparable between the maxilla and mandible. miRNA levels derived from the gingival crevicular fluid (GCF) of the 24 patients were compared before treatment start. Linear regression of individual data points is shown with 95% confidence interval. Relative miRNA levels are shown after log2-transformation and normalization. Pearson correlation coefficient is given. All *p* values ≤ 0.041.

**Fig. 2:**
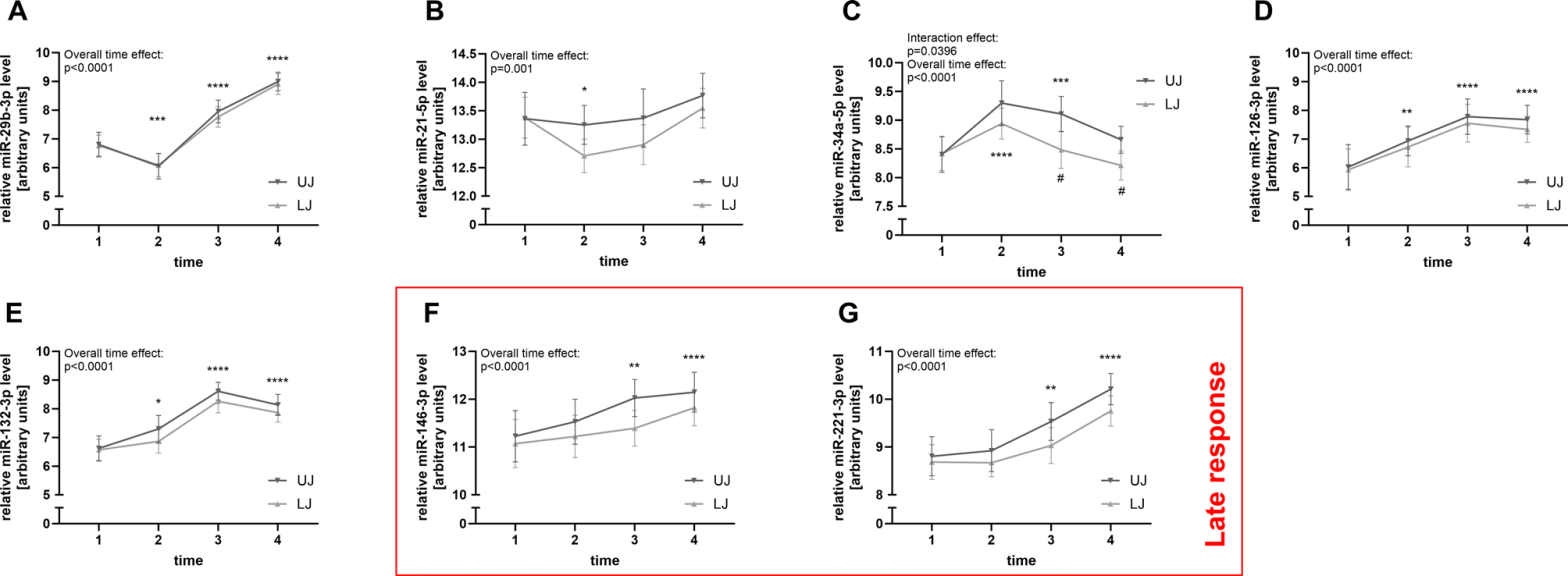
miRNA alterations during full-mouth multibracket appliance (MBA) treatment can be classified as early- and late-response. miRNA levels from the gingival crevicular fluid (GCF) of the 24 patients are presented by jaw (UJ, upper jaw [maxilla]; LJ, lower jaw [mandible]) at the respective visits: T1, pre-treatment assessment, T2, after one week, T3 after five weeks, T4 after three months. The red box indicates the late treatment response marked by increased levels of miR-146a-5p and miR-221-3p. Data is given as mean and 95% confidence interval. Mixed-model analysis was used to identify differences over time between the upper and lower jaw (interaction effect). Relative miRNA levels are shown after log2-transformation and normalization. Significant interaction effect (time × jaw) is indicated by ^#^; significant differences compared to pre-treatment level (T1) are indicated by asterisks, *, *p* < 0.05; **, *p* < 0.01; ***, *p* < 0.001; ****, *p* < 0.001.

To analyze if the levels of the examined miRNAs are comparable between the upper and lower jaw in general, miRNA expression levels before MBA application (baseline, T1), were compared. This analysis suggested moderate to high correlation between GCF samples taken from the mandible and maxilla with correlation coefficients between r = 0.42 and r = 0.71 (all p ≤ 0.041) (Fig. 1). Of note, the lowest correlation was detected for vascular miR-126, which might be based on the known vascularization differences between the lower and upper jaw ^45^. All seven analyzed miRNAs showed changes in their expression levels during orthodontic tooth movement compared to baseline (significant time effect, all *p* < 0.001) (Fig. 2). The general profile indicated an increase in miRNA expression levels in both jaws with time except for miR-21-5p, which showed slightly reduced expression levels at T2 one week after MBA application (*p* = 0.046). For miR-34-5p, a significant interaction effect was observed (time × jaw, *p* = 0.0396) in that lower levels were found after five weeks and three months of treatment in the mandible compared to the maxilla (Fig. 2).

**Fig. 3:**
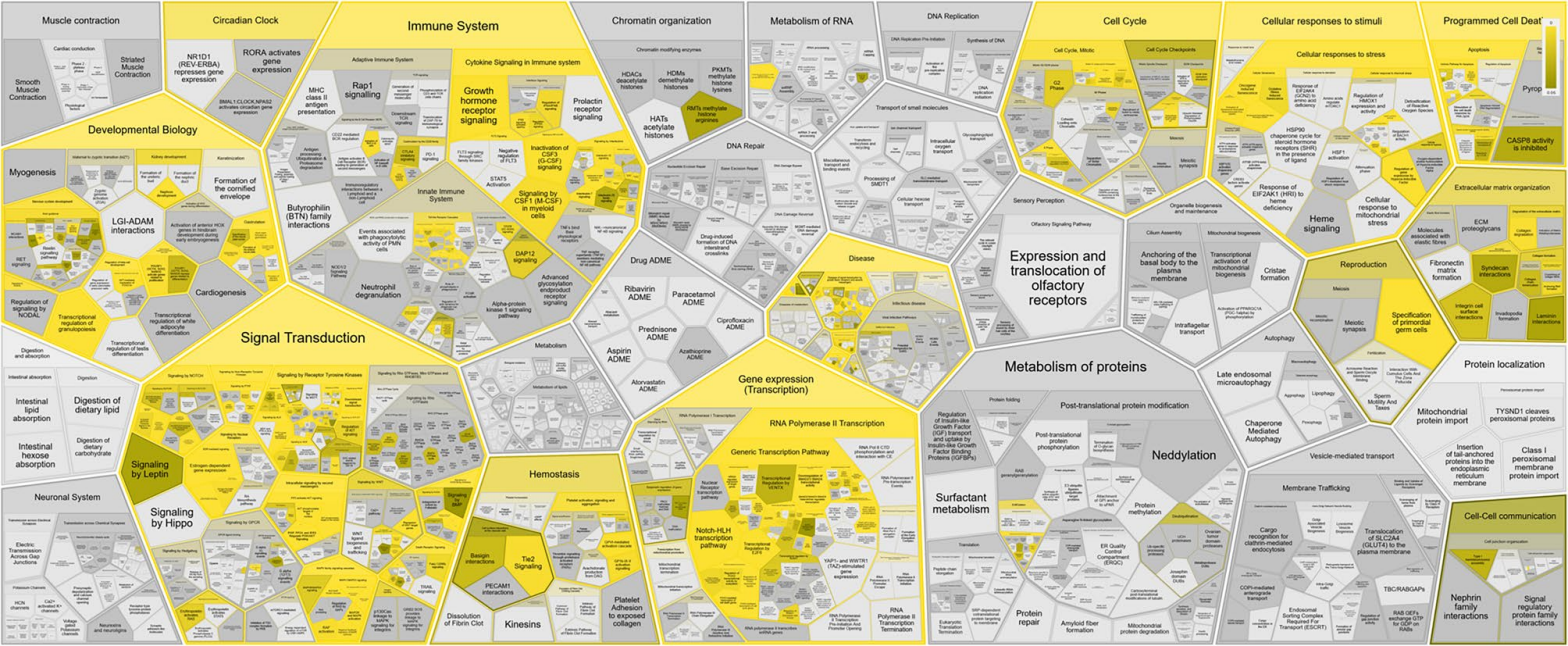
Pathways regulated by the analyzed miRNAs. Visualization of overrepresented pathways (yellow) was performed using the Reactome online analysis tool (Version 87, human targets). In vitro validated targets of miRNAs were submitted to determine pathway enrichment. Darker shades indicate lower *p* values.

The identified functionally validated target genes of regulated miRNAs are provided in Supplemental Table 1. Pathway analysis of the combined set of identified miRNA targets revealed several overrepresented pathways regulated by the identified miRNAs including cellular response to stress including hypoxia as well as extracellular matrix organization (Fig. 3). Comparison of pathways including targets regulated early during tooth movement with targets of the late-response miRNAs, miR-146 and miR-221 (Fig. 2), suggested that specifically during the medium to late phase of the treatment, regulation of NOD1/2 as well as RHOH GTPAse and FAS/CD95-L might occur (Supplemental Figs. 1 and 2).

## Discussion

This study aimed to identify a treatment-dependent profile of functional periodontal-inflammatory miRNAs during MBA application in healthy adolescent patients. Using subsequent target gene analysis, regulatory pathways induced by orthodontic tooth movement involved in bone resorption and apposition over time were identified. Our main findings are 1) miRNA levels determined in pre-treatment GCF samples are largely comparable between the mandible and maxilla, 2) while a clear time-dependent miRNA profile in both jaws over three months of MBA was observed, miR-34a-5p expression levels differed significantly between the mandible and maxilla starting after 5 weeks of treatment, 3) elevated levels of miR-146 and miR-221 were only observed during the medium to late phase of MBA application, and 4) a specific set of miRNA targets was identified indicating regulation of multiple targets including cellular response to stress including hypoxia as well as extracellular matrix organization. Moreover, it was shown that the applied approach allowed for the stable and reliable detection of miRNA levels based on non-invasive GCF samples.

Although miRNAs have long been discussed as important and ubiquitous regulators in bone metabolism, tissue regeneration, and as inflammatory mediators, only a limited number of studies on the effects of orthodontic forces on the miRNA composition in GCF and regulated pathways are available. To the best of our knowledge, our study is the first to describe a miRNA profile determined from GCF samples of both jaws during three months of MBA treatment in adolescent patients with subsequent analysis of functional targets and associated pathways. Our data can be interpreted in the light of known pathways involved in the remodeling process of alveolar bone. miR-21 has previously been shown to be upregulated in PDLCs of premolars after one month of orthodontic force application (80 – 100 g) ^28^, while we found a slight downregulation of miR-21 during the initial treatment phase. Regulation of miR-21 during the early phase of tooth movement may be explained by the observation that inhibiting miR-21 downregulates osteoclastogenesis and alveolar bone resorption during orthodontic tooth movement likely via the PDCD4 and IL-12A axis, regulating the chondroitin/ dermatan sulfate degeneration pathway ^46^. miR-29, a potential regulator of the extracellular matrix, has already been reported to be upregulated after 7 days of treatment (powerchain, ~ 250 g) in the GCF of adolescents during canine retraction and was found in both, exosome-depleted and non-depleted supernatants ^27^. This is partly in line with our results, even though we detected an initial downregulation after MBA application which may be explained by the lower force applied or the fact that no teeth were removed at the beginning of the treatment. Previous analyses suggested, that miR-29b targets different collagens (COL-1A1, -3A1, -4A1), as well as MMP2 and TGFβ2, all known to be regulated during matrix remodeling ^47,48^. In vitro experiments revealed that miR-29b inhibits TGFβ leading to increased RUNX2 levels and subsequently enhanced osteoblastogenesis and COL-1 expression. Furthermore, miRNA-29b is directly involved in the inhibition of COL-1 mRNA in the late phase of mineralization via interaction with its 3’UTR ^49^ Thus, miR-29b may potentially prevent bone fibrosis in the late stages of mineralization through COL-1 inhibition ^10,18,49^. It thus seems conceivable that miR-29b is downregulated in the early phase of orthodontic tooth movement, where the elimination of the hyaline phase and the resorption of alveolar bone by osteoclasts are prominent. In this phase, increased TGFβ expression dampens osteocytogenesis, and increased COL-1 expression in PDLCs contributes to the regeneration capacity of the PDL ^50^. Later in tooth movement, miR-29b levels increase, potentially activating the expression of RUNX2 through TGFβ inhibition, promoting osteoblast differentiation for bone formation and protecting against fibrosis by directly inhibiting COL-1. Another miRNA involved in matrix reorganization, miR-34a, has been reported to be downregulated during orthodontic tooth movement after 24 h for up to 4 weeks ^38^. This partly contradicts the results of the present study, where an initial upregulation was observed for up to 5 weeks. Of note, a significant difference in the expression levels of miR-34a over time was found between the upper and lower jaw, a previously unreported finding. On the molecular level, a negative correlation of miR-34 and MMP-2, -9, and -14 in PDLCs has been demonstrated, affecting matrix degradation and osteoclast differentiation, and thus resorption of alveolar bone ^38^. The target analysis for miR-34a revealed lactate dehydrogenase (LDH) A, which is present in the cell plasma and enters the extracellular matrix upon tissue destruction ^51^. An increased LDH level in the GCF, which has been demonstrated after force application to canine teeth of orthodontic patients ^52^, indicates increased inflammatory potential of the gingiva, which may potentially trigger a counter regulation of miR-34a. To this extent, miR-34a has been reported upregulated during osteoblast differentiation and miR-34a overexpression inhibited late osteoblast differentiation through LDHA-controlled cellular anaerobic glycolysis ^53^. It can be assumed that miR-34a inhibits late osteoblast differentiation through LDHA or LDA during orthodontic tooth movement, while affecting extracellular matrix degradation and osteoclast differentiation through MMPs. The observed expression differences between the upper and lower jaw could be attributed to the reported differences in bone density, PDL composition, or differential blood supply of the maxilla and mandible ^45^.

With respect to vascularization changes during orthodontic tooth movement, it is known that miR-126a plays a central role in vasculogenesis ^30^. Here, miR-126 was increased over the entire treatment period and target analysis identified known targets of miR-126, including chemokine receptor CXCR4 and VEGFA ^54,55^. Of note, miR-126 has been discussed as upregulated under hypoxic conditions ^30^ and overexpression of miR-126 in vitro has been shown to reduce the expression of IL-1α, IL-6, and TNFα, which are increased in the periodontium after orthodontic force application ^18^. Consistently, injection of miRNA-126 mimics into inflamed periodontal tissue in rats has led to reduced bone resorption and osteoclastogenesis ^10,17,56^. In addition to the anti-inflammatory effect, miRNA-126 may possibly prevent excessive angiogenesis and osteoclast recruitment by directly inhibiting VEGF. We also observed elevated miR-132 levels. While there is little evidence regarding miR-132 activity in the PDL or alveolar bone, it has been described that miR-132 expression in PDLCs is mechanosensitive and shear stress-dependent ^34^. Of note, targets of miR-132 include sirtuin 1 (SIRT1) ^57^, which regulates bone metabolism and bone mass ^58^, potentially involving an osteogenesis-inhibiting effect. Moreover, SIRT1 is expressed in the vasculature during blood vessel growth, controlling angiogenic activity ^59^.

During the late phase of the MBA treatment (> 5 weeks), miR-146 and -221 were upregulated. miR-146 has been shown to promote angiogenesis via a comparable route as miR-126, targeting the angiostatic chemokine CXCR4 ^54^. Of note, a study using mini-implant-supported canine retraction after premolar extraction (150 cN), also did not detect upregulation during the early phase of treatment ^31,32^, suggesting that miR-146 is induced as part of a late response in orthodontic tooth movement, potentially balancing the anti-angiogenic activity of miR-132. miR-146a may also be part of an anti-inflammtory response since the validated targets include IL-1-receptor-associated kinase-1 (IRAK1) ^60^, and IRAK1 downregulation may inhibit the proinflammatory cytokines IL-6, IL-8, and TNF-α. For miR-221, in vitro studies showed that miR-221 attenuated the bone-forming potential of osteoblasts, probably by downregulating TIMP-3 ^61^. Furthermore, platelet-derived growth factor A (PDGFA) was identified as a target of miRNA-221, which stimulates fibroblast proliferation during bone fracture healing, leading to improved bone regeneration ^62^. Comparison of the pathways regulated early during tooth movement and pathways regulated by the late-response miR-146a and -221 revealed that NOD1/2 as well as RhoH GTPAse and FAS/CD95 may be regulated specifically during this phase of orthodontic tooth movement. It has been reported that Rho family GTPases such as Rho and Rac are involved in actin assembly and stress fiber formation ^63^ and that different members of the Rho family are activated by tensile and compressive stress ^21^. A change in Rho family member regulation might thus indicate a difference in the predominant stressors over time. The FAS ligand (FASL) induces programmed cell death by building the death-inducing signaling complex (DISC) and the FAS ligand/receptor interactions thus play an important role in immune system regulation ^64^. Thus, an overactivation of the DISC in tooth movement might lead to the expression of miR-146a to prevent excessive tissue damage by apoptosis. The role of NOD receptors during tooth movements is currently not clear, however, NOD1 and 2 are intracellular pattern recognition receptors functionally expressed in PDLCs ^65^ and mediate innate and acquired immunity. It is thus conceivable that the upregulation of miR-146a and -221 during the later phase of MBA treatment controls NOD signaling to reduce inflammatory processes.

## Limitations

Even though this study had a relatively long observational period, changes in miRNA profiles beyond 3 months of orthodontic tooth movement may need to be investigated to provide a full picture of regulatory processes over the entire treatment period. Also, miRNA profiles in this study were investigated in adolescent patients. Thus, results may not be generalized to orthodontic tooth movement in adults where factors such as periodontitis are known to affect the miRNA profile. Severity of malocclusion in patients has not been evaluated. The effects of magnitude of movement or applied effective force on miRNA expression could not be determined.

## Conclusion and practical implications

We conclude that a specific periodontal-inflammatory profile of functional miRNAs involved in orthodontic tooth movement can be determined using non-invasive sampling of GCF. MBA treatment-induced changes in miRNA levels were time-dependent and largely comparable in both jaws, indicating an early and medium to late phase in tooth movement, which was marked by an increase of miR-146 and miR-221. The identified miRNA profile was linked to known targets involved in the remodeling process of the alveolar supporting apparatus and alveolar bone including cellular response to stress including hypoxia, vascularization, osteoclastogenesis as well as extracellular matrix organization. GCF-derived miRNAs may thus represent diagnostic biomarkers to monitor cellular processes induced by orthodontic tooth movement over time and could be used to optimize individual treatment outcomes. The local pharmacological modulation of the miRNAs identified in this study may represent an option to accelerate tooth movement leading to reduced treatment time. This option warrants further investigation in future studies.

## Supplementary Information

Supplementary Information 1.

Supplementary Information 2.

## Data Availability

The datasets generated during and/or analysed during the current study are available from the corresponding author on reasonable request.
