# Supplementary material for: Characterization of a periodontal-inflammatory microRNA profile during multibracket orthodontic treatment in adolescents
Source: Sci Rep. 2025 Jun 3;15:19488. doi: 10.1038/s41598-025-01794-6 (PMC12134260; doi:10.1038/s41598-025-01794-6)
Supplement: Supplementary file 1 — Supplementary Information 1. [file 41598_2025_1794_MOESM1_ESM.pdf]

Supplement

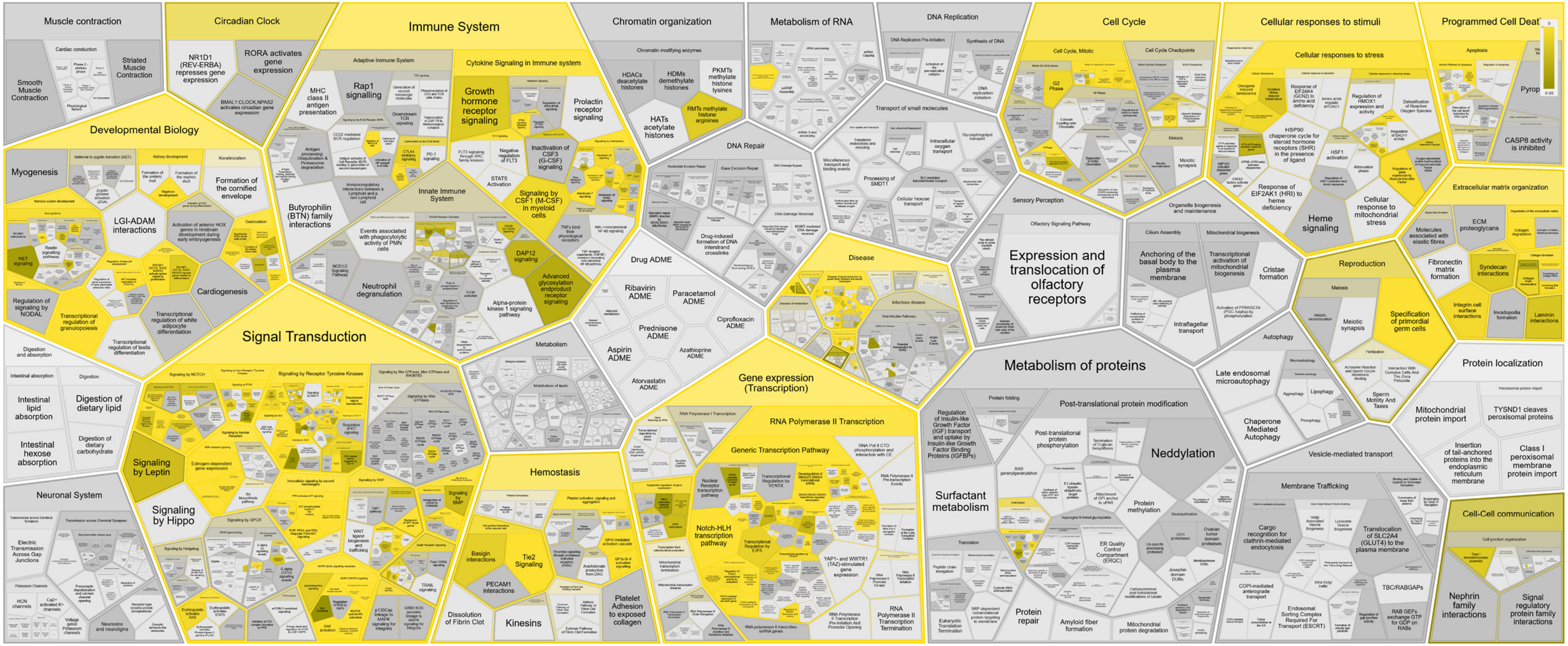

Supplemental Figure 1: Early-onset pathways. Visualization of overrepresented pathways (yellow) was performed using the Reactome online analysis tool (Version 87, human targets). *In vitro* validated targets of miRNAs miR-21-5p, miR-29b-3p, miR-34a-5p, miR-126-3p, and miR-132-3p were submitted to determine pathway enrichment. Darker shades indicate lower p values.

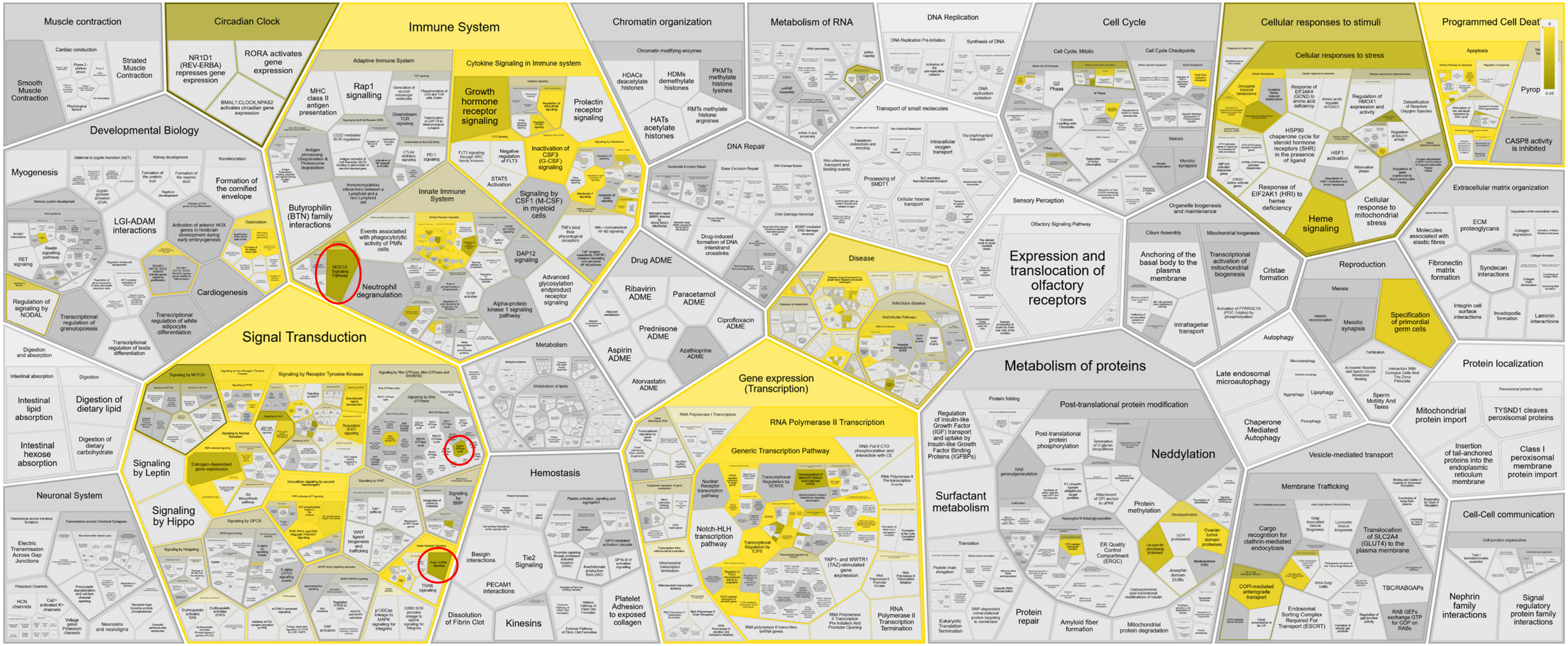

Supplemental Figure 2: Late-onset pathways. Visualization of overrepresented pathways (yellow) was performed using the Reactome online analysis tool (Version 87, human targets). *In vitro* validated targets of miRNAs miR-146a-5p and miR-221-3p were submitted to determine pathway enrichment. NOD1/2, RHOH, and FASL/CD95L are marked by red circles. Darker shades indicate lower p values.
